# Field study examining the mucosal microbiome in equine glandular gastric disease

**DOI:** 10.1371/journal.pone.0295697

**Published:** 2023-12-07

**Authors:** Linda J. Paul, Aaron C. Ericsson, Frank M. Andrews, Zachary McAdams, Michael L. Keowen, Michael P. St Blanc, Heidi E. Banse

**Affiliations:** 1 Veterinary Clinical Sciences, Equine Health and Sports Performance, Louisiana State University, Baton Rouge, LA, United States of America; 2 Metagenomics Center, Equine Gut Group, University of Missouri, Columbia, MO, United States of America; Texas A&M University College Station, UNITED STATES

## Abstract

Equine glandular gastric disease (EGGD) is a common disease among athletic horses that can negatively impact health and performance. The pathophysiology of this EGGD remains poorly understood. Previous studies using controlled populations of horses identified differences in the gastric glandular mucosal microbiome associated with disease. The objective of this study was to compare the gastric microbiome in horses with EGGD and those without across multiple barns and differing management practices. We hypothesized that alterations in the microbiome of the gastric glandular mucosa are associated with EGGD. A secondary objective was to perform a risk factor analysis for EGGD using the diet and management data collected. Microbial populations of biopsies from normal pyloric mucosa of horses without EGGD (control biopsies), normal pyloric mucosa of horses with EGGD (normal biopsies) and areas of glandular mucosal disruption in horses with EGGD (lesion biopsies) were compared. Lesion biopsies had a different microbial community structure than control biopsies. Control biopsies had a higher read count for the phylum *Actinomycetota* compared to lesion biopsies. Control biopsies also had an enrichment of the genera *Staphylococcus* and *Lawsonella* and the species *Streptococcus salivarius*. Lesion biopsies had an enrichment of the genera *Lactobacillus* and *Actinobacillus* and the species *Lactobacillus equigenerosi*. These results demonstrate differences in the gastric glandular microbiome between sites of disrupted mucosa in horses with EGGD compared to pyloric mucosa of horses without EGGD. Risk factor analysis indicated that exercise duration per week was a risk factor for EGGD.

## Introduction

Equine glandular gastric disease (EGGD) is a common disease among athletic horses that can negatively affect health and performance. Prevalence data for EGGD varies based on breed, discipline, and competition status, but can be as high as 70% [1–4]. Horses diagnosed with EGGD have owner-reported complaints that include poor performance (65%), changes in behavior (33%), weight loss/poor body condition (87%), and recurrent colic (74%) [5, 6]. While the pathophysiology of equine squamous gastric disease (ESGD) has been primarily linked to excessive acid exposure, the underlying mechanisms of EGGD remain poorly understood. Response of EGGD to traditional antacid therapy with proton pump inhibitors (PPI) is relatively poor compared to the response of ESGD to this treatment [7]. This difference in response to acid suppression indicates a more complex pathophysiology of EGGD compared to ESGD. Mechanisms that have been investigated as contributing factors to EGGD include decreased protective factors such as decrease in prostaglandins with administration of non-steroidal anti-inflammatory drugs (NSAIDs) [8, 9], decreased blood flow with exercise [10], response to stress [11], inflammation [12], and bacterial colonization [13–18].

*Helicobacter pylori* is commonly associated with gastritis in people but does not appear to be related to EGGD [13–17]. In non-*H*. *pylori* gastritis in people, alterations of the gastric mucosal microbiota was demonstrated [19]. Previous studies in horses have also found evidence to support differences in community structure of the gastric glandular mucosal microbiome in association with EGGD lesions [18, 20]. These studies had limited numbers of horses, and sampled horses from a single location. The gastric microbiome appears to vary substantially among horses, especially with different diet and management strategies [13, 14, 21]. We hypothesized that alterations in the microbiome of the gastric glandular mucosa are associated with EGGD. The primary objective of this study was to compare the gastric mucosal microbiome in horses with and without EGGD across multiple barns and differing management practices to determine if differences persist in a larger, more varied population. A secondary objective was to perform a risk factor analysis for EGGD using the diet and management data collected.

## Materials and methods

This data was collected as a case-control study in horses with naturally occurring EGGD. The study protocol was approved by the Louisiana State University Institutional Animal Care and Use Committee (LSU IACUC 19–071). Client-owned horses involved in athletic performance were recruited from the southern Louisiana region. Owner consent was obtained prior to enrollment and a questionnaire completed as previously described [21]. All gastroscopic evaluations were performed by the same licensed veterinarian and board-certified internal medicine specialist (HEB) (S1 File). After an overnight fast, horses underwent sedation (xylazine, 0.3–0.4 mg/kg IV) for gastroscopic evaluation with a 3 m endoscope. Presence of EGGD was recorded using a semi-quantitative scale and descriptive terms [7, 22]. Only horses with no evidence of glandular disease (control group; EGGD score = 0) or horses with glandular mucosal disruption (affected group; EGGD score ≥2) were enrolled. Horses with hyperemia as the only glandular mucosal abnormality (EGGD score = 1) were excluded.

### Sample collection

Pinch biopsy forceps (2.3 mm oval cupped with spike) were used to collect mucosal biopsies of endoscopically normal (median number of biopsies collected = 3; range 1–4) and areas of glandular mucosal disruption (median = 2; range 1–3) in horses with and without EGGD. Biopsies of normal mucosa were collected from the pylorus. Biopsies were rinsed with sterile saline then flash frozen in liquid nitrogen and stored at -80°C until processing. The biopsy channel and biopsy forceps were disinfected between horses with an accelerated hydrogen peroxide product (Rescue, Virox, Technologies Inc., Oakville, ON, Canada) to prevent microbial cross contamination. Sterile water was used to rinse these instruments after disinfection.

### DNA extraction and sequencing

DNA was extracted using a commercial kit from gastric mucosal biopsies, according to the manufacturer’s recommendations, with minor adaptations as previously described [23]. The 16S rRNA amplicon library construction and sequencing were performed at the University of Missouri DNA Core facility. Concentrations of DNA for each sample were determined fluorometrically using quant-iT BR dsDNA reagent kits and all samples normalized to a standard concentration for PCR amplification. Bacterial 16S rRNA amplicons were generated via amplification of the V4 hypervariable region of the 16S rRNA gene using single-indexed universal primers (U515F/806R) flanked by Illumina standard adapter sequences and the following parameters: 98°C^(3:00)^+[98°C^(0:15)^+50°C^(0:30)^+72°C^(0:30)^] × 25 or 40 cycles +72°C^(7:00)^. Amplicons were then pooled for sequencing using the Illumina MiSeq platform and V2 chemistry with 2×250 bp paired-end reads, as previously described [24].

All 16S rRNA amplicon sequence processing was performed using Quantitative Insights Into Microbial Ecology 2 (QIIME2) v2021.4 [25]. Primers designed to match the 5’ ends of forward and reverse reads were removed from the forward read using Cutadapt [26]. Reverse complements of the primer to any reverse reads present were removed from the forward read. For reverse reads, a similar, but opposite approach was performed. Read pairs were rejected if either did not match a 5’ primer with an allowable error rate of 0.1. Untrimmed sequences were discarded. To denoise, de-replicate, and count amplicon sequence variants (ASVs), the QIIME2 [25] DADA2 plugin [27] were utilized. Biopsy samples returning >5,000 ASV read counts were rarefied to 5,000 reads and included in microbiome comparisons. Final taxonomies were assigned using the SILVA 138 [28] reference database trimmed to the V4 region (S2 File).

### Statistical analysis

Signalment data (age, breed, sex, discipline) were compared between control and affected groups using the Mann-Whitney U test or Chi-Square test.

Repetitive biopsy samples meeting inclusion criteria were averaged. Samples were compared based on the endoscopic appearance of mucosal biopsy site: control samples (CON-BX) from pyloric glandular mucosa in horses with no evidence of EGGD, normal samples (NORM-BX) from endoscopically normal pyloric glandular mucosa in horses with EGGD ≥2, and lesion samples (LES-BX) from areas of disrupted glandular mucosa in affected horses with EGGD≥2 (S3 File). Independent sample comparisons were made for CON-BX vs. NORM-BX and CON-BX vs. LES-BX. Paired sample comparisons were made for NORM-BX vs. LES-BX. Overall compositional differences associated with EGGD status were assessed using similarity (β-diversity) indices of ASV data. One-way permutational multivariate analysis of variance (PERMANOVA) was used with 9,999 permutations [29]. Jaccard and Bray-Curtis similarity indices were used to assess for compositional differences based on what taxa are present (unweighted) as well as for the effect of taxa present in combination with the abundance of those taxa (weighted), respectively [29]. Principal coordinate analysis (PCoA) was performed on ¼ root-transformed rarefied data [29]. The α-diversity indices (diversity within subjects) Taxa_S (TS), Shannon_H (SH), and Chao-1 (C1) of ASV data were calculated [29] and values compared among groups via Mann-Whitney U or Wilcoxon Signed Rank test [30]. Significance was set at p-value ≤ 0.05.

Direct comparison of read counts for taxa stratified to phylum (L2), genus (L6), and species (L7) levels were performed between groups using Mann-Whitney U test or Wilcoxon Signed Rank test [30] followed by Benjamini-Hochberg procedure with a false discovery rate (FDR) of 5%. Linear discriminant analysis (LDA) effect size (LEfSe) was used to identify taxa enrichment associated with biopsy groups. Significance was set at adjusted p-value ≤ 0.05 and LDA score ≥ 2 [31, 32].

Risk factor analysis for EGGD based on diet and management variables was also assessed using backwards binomial logistic regression. As data were not normally distributed, continuous variables were dichotomized at the median (age, number of years competing, number of years at current barn, and minutes per week of exercise). Variables were assessed with univariate analysis (Chi-Square or Fisher’s Exact) and those with a p-value < 0.1 considered for the binomial logistic regression. Collinearity was assessed (Spearman’s correlation) and considered present if correlation coefficients were > 0.7. When collinearity was identified, multiple models were constructed and only one of the collinear variables was included. Backward elimination was used to remove variables with p > 0.05. Confounding was assessed by observing changes > 20% in the remaining coefficients. Akaike information criterion (AIC) was assessed and the model with the lowest AIC retained.

## Results

Fifty-seven horses from ten barns were enrolled in this study (controls, n = 25; affected, n = 32). The median age of the horses was 5 years (range: 1–20; n = 53). There was no difference detected in age (*p* = 0.368), sex (*p* = 0.507; females, n = 21; males, n = 33), breed (*p* = 0.184; TB, n = 37; non-TB, n = 17), or discipline (*p* = 0.197; race, n = 22; show, n = 35) between control and affected horses. Further details on the study population and the influence of diet and management strategies on the gastric microbiome were previously reported [21]. All horses with areas of abnormal mucosa had lesions within the pylorus/antrum (n = 32). Three horses had additional lesions in the glandular fundus. Endoscopic lesion phenotypes were flat fibrinosuppurative (n = 13), flat hemorrhagic (n = 5), flat fibrinosuppurative + hemorrhagic (n = 7), raised fibrinosuppurative (n = 4), raised fibrinosuppurative + hemorrhagic (n = 1), flat hemorrhagic and raised fibrinosuppurative (n = 1), and flat fibrinosuppurative + hemorrhagic and raised fibrinosuppurative (n = 1).

### Microbiome community comparisons

Beta diversity differed between CON-BX and LES-BX using both Jaccard (*p* = 0.031) and Bray-Curtis (*p* = 0.002) similarity indices. Between CON-BX and NORM-BX, there was a difference detected using the Bray-Curtis similarity index (*p* = 0.010), but not with the Jaccard index (*p* = 0.217). There was no difference in beta diversity detected between LES-BX and NORM-BX (Jaccard, *p* = 1.000; Bray-Curtis, *p* = 0.957) (Table 1).

**Table 1:** Comparison of beta-diversity PERMANOVA results between biopsy types.

	CON-BX v. LES-BX	CON-BX v. NORM-BX	NORM-BX v. LES-BX
Jaccard Index			
p-value	0.031*	0.217	1.000
F-value	1.223	1.070	0.676
Bray-Curtis Index			
p-value	0.002*	0.010*	0.957
F-value	2.409	1.911	0.634

CON-BX, control pyloric glandular mucosal biopsies from horses without equine glandular gastric disease (EGGD); LES-BX, biopsies from areas of glandular mucosal disruption in horses with EGGD; NORM-BX, biopsies of endoscopically normal pyloric glandular mucosa in horses with EGGD. * indicates p-value ≤ 0.05

**Fig 1:**
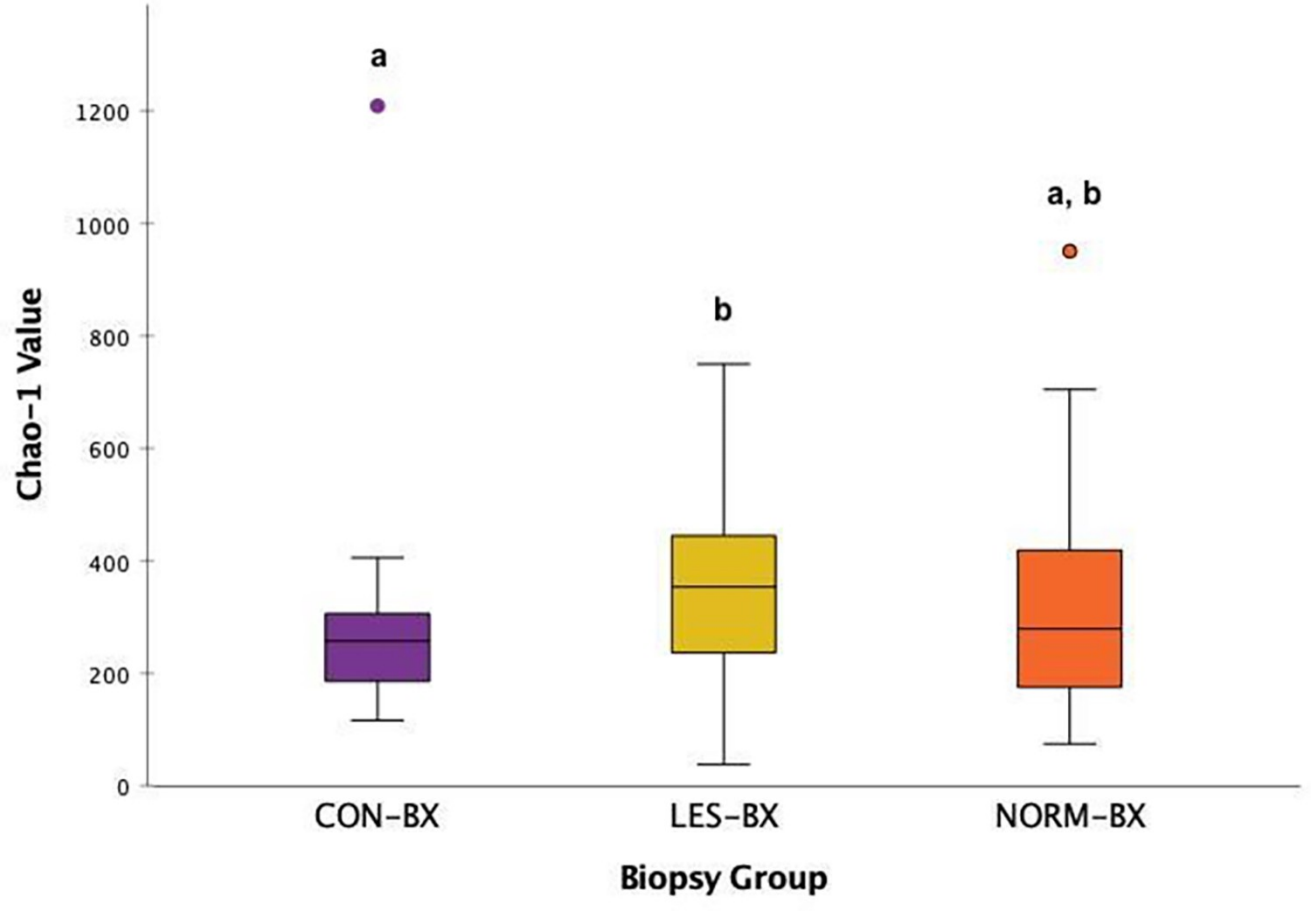
Comparison of alpha-diversity Chao-1 values between biopsy groups. Boxplot depiction of Chao-1 values for pyloric glandular mucosal biopsies in horses without evidence of equine glandular gastric disease (EGGD) (CON-BX), biopsies from areas of glandular mucosal disruption in horses with EGGD (LES-BX), and endoscopically normal pyloric glandular mucosa in horses with EGGD (NORM-BX). Different letter superscripts indicate p-value ≤ 0.05.

Alpha diversity differed between CON-BX and LES-BX using the Chao-1 richness index (p = 0.037, Fig 1), but not with the use of Taxa S (*p* = 0.063) or Shannon index (*p* = 0.917). There was no difference detected in alpha diversity between CON-BX and NORM-BX (Chao-1, *p* = 0.574; Shannon, *p* = 0.664; Taxa S, *p* = 0.688) or between LES-BX and NORM-BX (Chao-1, *p* = 0.125; Shannon, *p* = 0.575; Taxa S, *p* = 0.140).

No visual patterns were apparent on assessment of Principal Coordinate Analysis (PCoA) for comparisons between biopsy groups (S1 Fig).

### Individual taxa comparisons

**Fig 2:**
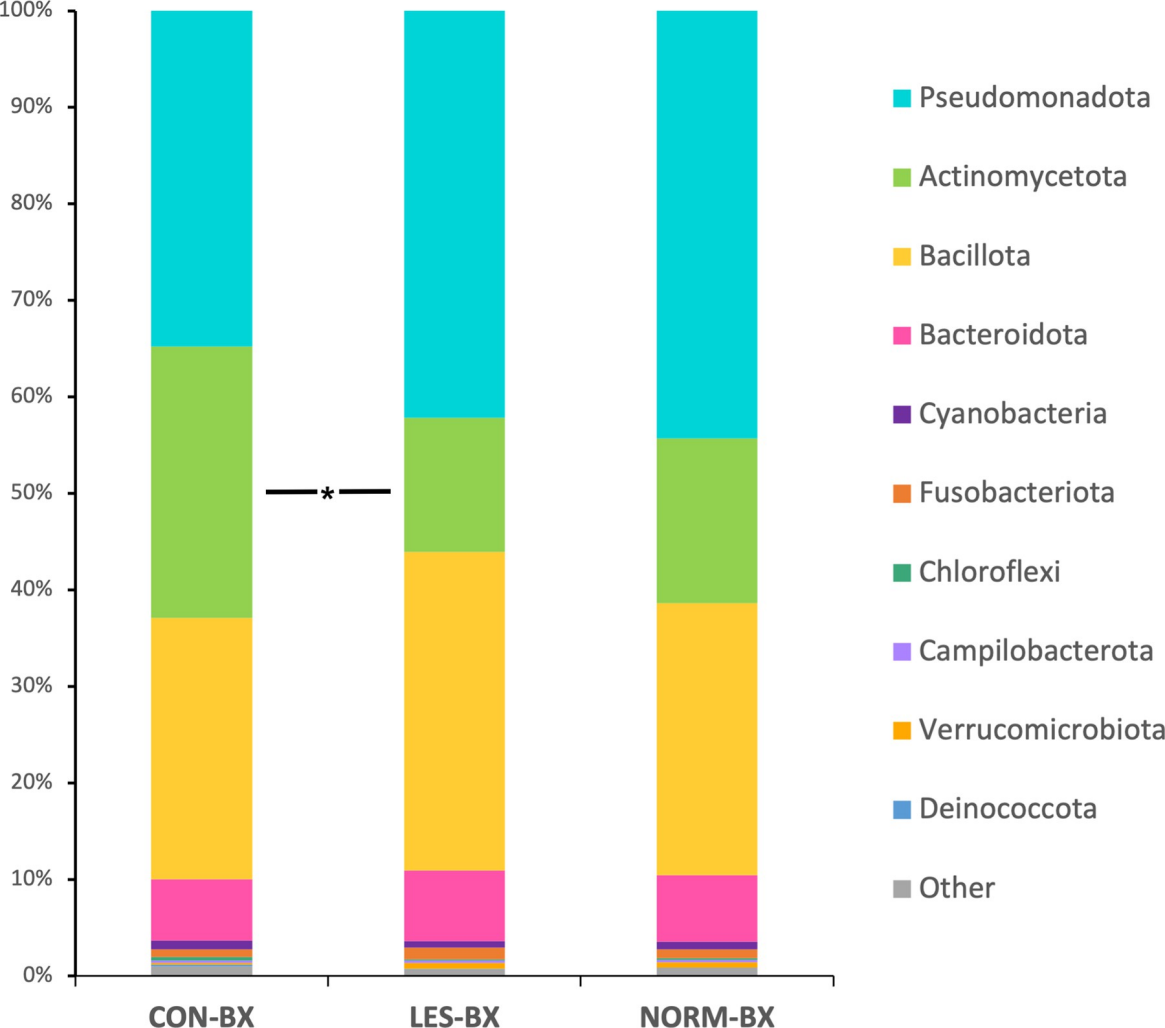
Stacked bar chart of major phyla for biopsy groups. Relative abundances of major phyla for pyloric glandular mucosal biopsies in horses without evidence of equine glandular gastric disease (EGGD) (CON-BX), biopsies from areas of glandular mucosal disruption in horses with EGGD (LES-BX), and endoscopically normal pyloric glandular mucosa in horses with EGGD (NORM-BX). * indicates significant difference based on 5% FDR of direct comparisons.

The major phyla detected in CON-BX were *Pseudomonadota*, formerly *Proteobacteria* [33], (35%), *Actinomycetota*, formerly *Actinobacteriota* and *Actinobacteria* [33, 34], (28%), and *Bacillota*, formerly *Firmicutes* [33], (27%). For LES-BX and NORM-BX, the major phyla detected were *Pseudomonadota* (42% and 44%, respectively), *Bacillota* (33%; 28%), and *Actinomycetota* (14%; 17%) (Fig 2). With direct comparisons, there were higher read counts for *Actinomycetota* in CON-BX compared to LES-BX (*p* = 0.001).

The major genera detected in CON-BX were *Actinobacillus* (14%), *Staphylococcus* (11%), *Cutibacterium* (10%), and *Streptococcus* (5%). Major genera of LES-BX were *Actinobacillus* (24%), *Enterococcus* (5%), *Sarcina* (5%), *Streptococcus* (5%), and *Staphylococcus* (5%). Major genera of NORM-BX were *Actinobacillus* (21%), *Cutibacterium* (6%), and *Streptococcus* (5%). No differences were detected in direct comparisons of read counts for genera among biopsy groups.

**Fig 3:**
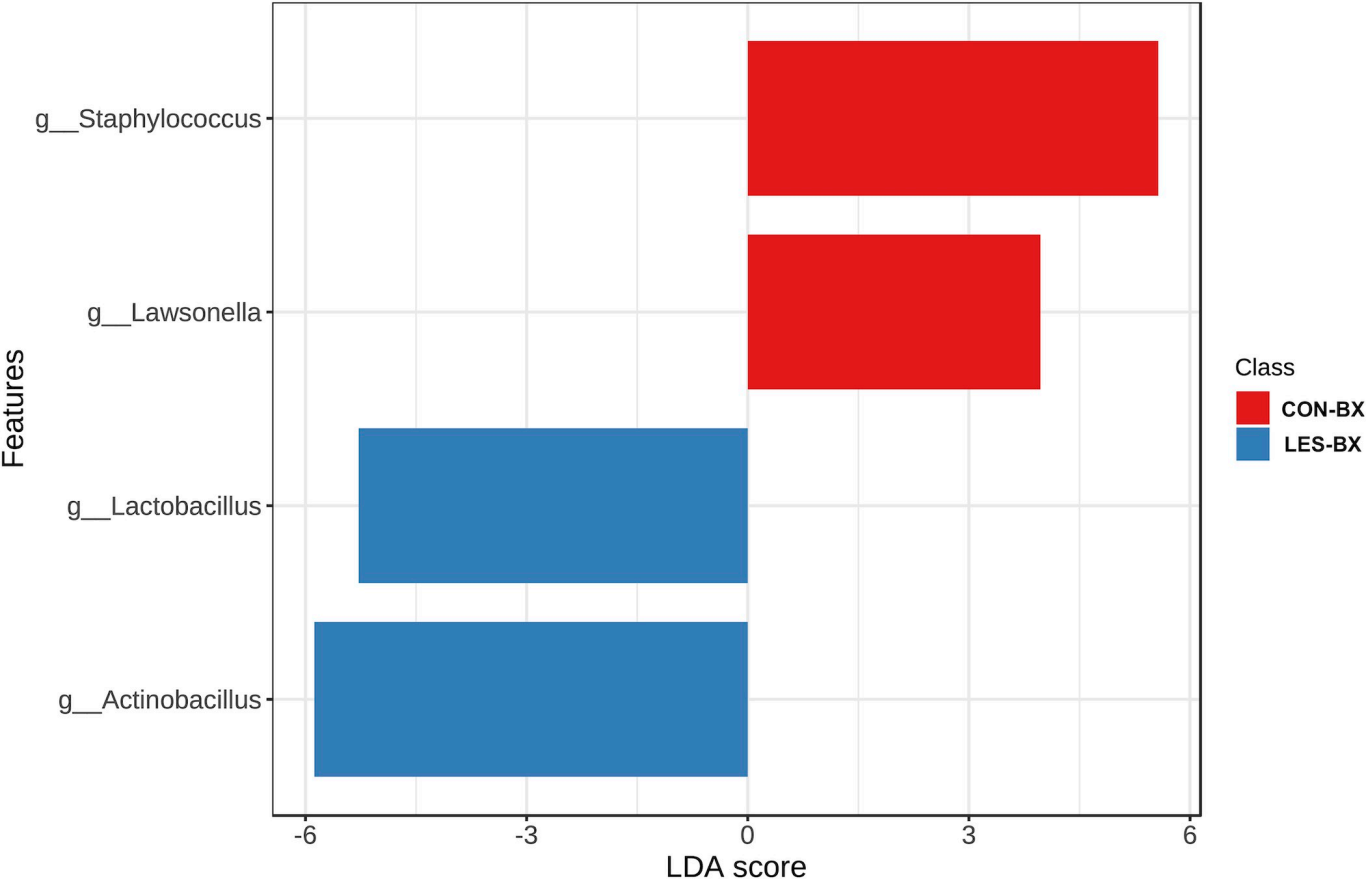
Linear discriminant analysis effect size (LEfSe) bar plot. Plot of genera significantly enriched (adjusted p-value ≤ 0.05 and LDA score ≥ 2) between pyloric glandular mucosal biopsies in horses without evidence of equine glandular gastric disease (EGGD) (CON-BX) and biopsies from areas of glandular mucosal disruption in horses with EGGD (LES-BX).

Linear discriminant analysis effect size (LEfSe) detected 4 genera significantly enriched between CON-BX and LES-BX (Fig 3). *Staphylococcus* (adjusted *p*-value = 0.04; LDA score = 5.57) and *Lawsonella* (adjusted *p*-value = 0.04; LDA score = 3.97) were enriched in CON-BX compared to LES-BX. *Lactobacillus* (adjusted *p*-value = 0.04; LDA score = -5.28) and *Actinobacillus* (adjusted *p*-value = 0.05; LDA score = -5.88) were enriched in LES-BX compared to CON-BX. To further investigate these taxa enrichments, LEfSe was performed at the species level. Enrichment of *Streptococcus salivarius* (adjusted *p*-value = 0.04; LDA score = 4.92) was detected in CON-BX and of *Lactobacillus equigenerosi* in LES-BX (adjusted *p*-value < 0.01; LDA score = -4.74). No taxa enrichment was detected between CON-BX and NORM-BX or NORM-BX and LES-BX.

### EGGD risk factor analysis

Variables included in the binomial logistic regression model were: hay in the diet (yes or no, Fisher’s exact = 0.072), alfalfa in the diet (yes or no, Fisher’s exact = 0.032), sweet feed in the diet (yes or no, Chi-Square *p* = 0.091), turnout provided (yes or no, Fisher’s exact = 0.022), stall containment as part of housing routine (yes or no, Fisher’s exact = 0.069), minutes per week of exercise (dichotomized at 180 minutes, Chi-Square *p* = 0.053). Collinearity was present between alfalfa in the diet and sweet feed in the diet (r_s_ = -0.786). Minutes per week of exercise was the only variable retained in the final model [*p* = 0.031, Exp(B) = 5.33 (95% confidence interval: 1.16–24.47)].

## Discussion

In the study presented here, modest differences in the bacterial microbiome were detected between pyloric glandular mucosal biopsies in horses without EGGD (CON-BX) compared to biopsies of disrupted glandular mucosa in horses with EGGD (LES-BX). There were differences detected in both beta and alpha diversity indices between these biopsy groups. Differences were also detected at the phylum, genus, and species level. At the phylum level, there was an increased number of reads for *Actinomycetota* in CON-BX compared to LES-BX. There were also enrichment differences for four genera and two species between these biopsy groups. Prior studies have identified mucosal microbial differences associated with EGGD in small populations from a single location [18, 20]. This study expands upon those findings by demonstrating comparable results in a larger, more diverse population of horses from several locations.

The use of Jaccard (unweighted) and Bray-Curtis (weighted) beta diversity indices detected differences between CON-BX and LES-BX. These results are similar to a prior study where the most substantial differences were between control horses (EGGD 0) and horses with mucosal disruption (EGGD ≥2) [20]. The higher alpha diversity Chao-1 value of LES-BX compared to CON-BX indicates an increased richness (higher number of taxa in the community) within the areas of disrupted mucosa. The chronology of when these differences occur (prior to or after onset of EGGD lesions) remains unknown. A difference in beta diversity (Bray-Curtis index only) was detected between CON-BX and NORM-BX. There were no differences detected in alpha or beta diversity between LES-BX and NORM-BX. The spectrum of findings across the comparisons between biopsy sites suggests the potential for a progressive change in the mucosal microenvironment among horses with and without disease, specifically at the site of lesions.

When examining enrichment of specific taxa across biopsy types with the use of LEfSe, enrichments were detected in the comparison of CON-BX and LES-BX. Control biopsies had an enrichment of the genera *Staphylococcus* (belonging to phylum *Bacillota*) and *Lawsonella* (belonging to phylum *Actinomycetota*) and of the species *Streptococcus salivarius* (belonging to phylum *Bacillota*). Lesion biopsies had an enrichment of the genera *Lactobacillus* (belonging to phylum *Bacillota*) and *Actinobacillus* (belonging to phylum *Pseudomonadota*) and of the species *Lactobacillus equigenerosi*. Previous studies have identified increases in *Bacillota* (reported as *Firmicutes*) associated with lesions in horses with EGGD and in humans with non-*H*.*pylori* antral gastritis [18, 19]. However, this study identified enrichment of different genera and species within the *Bacillota* phylum associated with CON-BX and LES-BX. An increased relative abundance of *Pseudomonadota* in normal mucosa compared to lesions has also been reported, [18] but in the present study, *Actinobacillus*, a genus within *Pseudomonadota*, was found to be enriched in LES-BX compared to CON-BX. The difference in results could reflect the different geographical regions of the study populations as well as the differences in study design including sample size, sampling techniques, and biopsy group designations. This highlights the complexity of EGGD and suggests that multiple factors may influence the gastric mucosal microbiome in disease.

*Lactobacillus*, and specifically *L*. *equigenerosi*, were enriched in LES-BX compared to CON-BX. Previous studies in equids have identified that the properties of *Lactobacillus* species are host-specific [14, 35] and identified *L*. *equigenerosi* as a component of the equine fecal microbiome [36, 37]. In people with gastric cancer, an overgrowth of *Lactobacillus* has been identified, although the implications of this have yet to be elucidated [38]. A previous study in horses identified *Lactobacillus* and *Streptococcus* adhered to the gastric glandular mucosal surface and deeply invasive at sites of disruption [14]. The clinical significance of the taxa differences between control and lesion biopsies identified in the present study and their potential role in EGGD remains unknown.

When examining potential diet and management risk factors in this study, horses with greater than 180 minutes of exercise per week were identified to have an increased risk of EGGD diagnosis. This supports other studies that have identified exercising 5–7 days per week [39] or 6–7 days per week [1] as a risk factor for EGGD. In people, gastrointestinal syndrome (GIS) occurs in both elite and recreational athletes and includes any gastrointestinal disturbance or dysfunction associated with exercise [40]. Exercise has not been associated with alterations in the equine gastric microbiome, suggesting other mechanisms may contribute to its role in EGGD. A decrease in blood flow to the gastrointestinal tract has been documented in people [41] and horses [10] which can make these organs, including the gastric glandular stomach, more susceptible to dysfunction and disease. Diet and management factors have previously been identified to alter the gastric microbiome [13, 14, 21]. However, none of these factors were retained in the final model as a risk factor for EGGD in the present study.

Limitations of this study include small biopsy mass, limited variation in study population, and a convenience sample of horses. The small biopsy mass may have limited our ability to successfully sequence the true mucosal microbiome, however, this was mitigated with visualization of rarefaction curves and the inclusion criteria of samples with greater than 5,000 reads for analysis. A recent study has also shown the use of cytology brushes as an alternative to biopsy samples for gastric glandular microbiome analysis [18]. The variation in study population was limited by the horses available in the region and were a convenience sample based on horse availability and owner consent.

## Conclusion

Differences exist in the gastric glandular mucosal bacterial microbiome between horses with EGGD and horses without endoscopic evidence of EGGD. The differences detected indicate a spectrum of changes in the mucosal microbiome between horses without EGGD, normal-appearing mucosa in horses with EGGD, and sites of disrupted mucosa in horses with EGGD. It is unknown if the changes in microbiome precede or follow onset of disease, but regardless of the chronology, the bacteria associated with glandular lesions might be influencing disease. Further studies are needed to assess if these bacteria are beneficial or contributing to onset or persistence of disease. Additionally, this study identified exercise as a risk factor for EGGD, although likely unrelated to changes in the microbiome. By better understanding the role of bacteria in EGGD and exercise as a risk factor, methods to manipulate the gastric microbiome and mitigate risk in highly active horses could lead to improved prevention and treatment strategies of this common and complex disease.

## Supporting information

S1 Fig Principal Coordinate Analysis (PCoA) plots. Principal Coordinate Analysis (PCoA) plots using (A) Jaccard and (B) Bray-Curtis indices of CON-BX and LES-BX.
(TIF)

S1 File Management factors. File containing the diet and management factors for each subject.
(XLSX)

S2 File Sample ASV data. File containing the amplified sequence variant (ASV) data for each sample.
(XLSX)

S3 File Sample ID key. File containing sample identification (ID) and subject information.
(XLSX)
