# Supplementary figures and images for: Field study examining the mucosal microbiome in equine glandular gastric disease
Source: PLoS One. 2023 Dec 7;18(12):e0295697. doi: 10.1371/journal.pone.0295697 (PMC10703338; doi:10.1371/journal.pone.0295697)

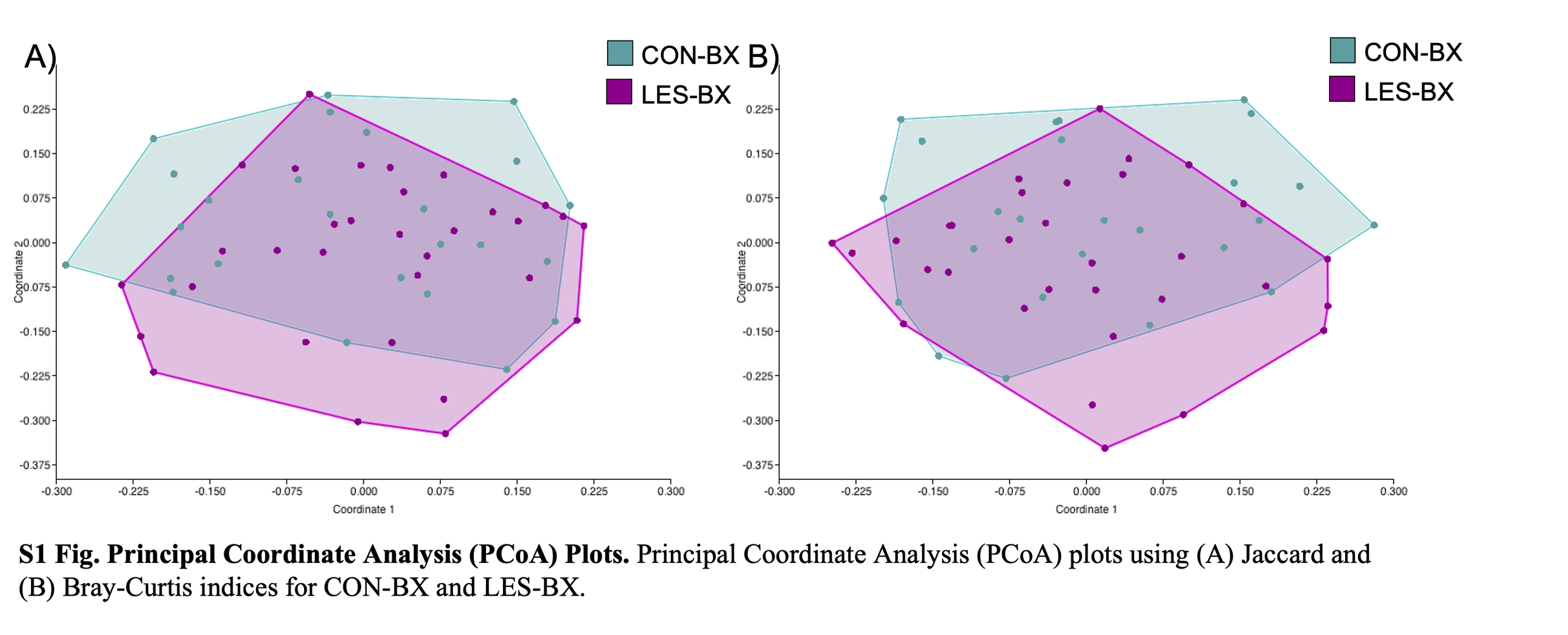

Supplement: S1 Fig — Principal Coordinate Analysis (PCoA) plots using (A) Jaccard and (B) Bray-Curtis indices of CON-BX and LES-BX. (TIF) [file pone.0295697.s001.tif]
